# The anti-influenza M2e antibody response is promoted by XCR1 targeting in pig skin

**DOI:** 10.1038/s41598-017-07372-9

**Published:** 2017-08-09

**Authors:** Charlotte Deloizy, Even Fossum, Christophe Barnier-Quer, Céline Urien, Tiphany Chrun, Audrey Duval, Maelle Codjovi, Edwige Bouguyon, Pauline Maisonnasse, Pierre-Louis Hervé, Céline Barc, Olivier Boulesteix, Jérémy Pezant, Christophe Chevalier, Nicolas Collin, Marc Dalod, Bjarne Bogen, Nicolas Bertho, Isabelle Schwartz-Cornil

**Affiliations:** 1 VIM-INRA-Université Paris-Saclay, Domaine de Vilvert, 78350 Jouy-en-Josas, France; 2 K.G. Jebsen Center for Influenza Vaccine Research, University of Oslo and Oslo University Hospital, 0027 Oslo, Norway; 3 Center for Immune Regulation, Institute of Immunology, University of Oslo and Oslo University Hospital Rikshospitalet, 0424 Oslo, Norway; 4 Vaccine Formulation Laboratory, University of Lausanne, Chemin des Boveresses 155, 1066 Epalinges, Switzerland; 5 UE1277-INRA, Plate-Forme d’Infectiologie Expérimentale - PFIE, 37380 Nouzilly, France; 6 Aix Marseille Univ, CNRS, INSERM, CIML, Centre d’Immunologie de Marseille-Luminy, 13288 Marseille, France; 7 GenoSafe, 1 bis rue de l’International, 91000 Evry, France; 8 Biostatistics, Biomathematics, Pharmacoepidemiology and Infectious Diseases (B2PHI), Inserm, UVSQ, Institut Pasteur, Université Paris-Saclay, 78180 Montigny-le-Bretonneux, France; 9 Genfit, 885 Avenue Eugène Avinée, 59120 Loos, France; 10 CEA - Université Paris Sud 11 - INSERM U1184, Immunology of Viral infections and Autoimmune Diseases (IMVA), IDMIT infrastructure, 92265 Fontenay-aux-Roses, France; 11 DBV Technologies, 177-181 avenue Pierre Brossolette, 92120 Montrouge, France

## Abstract

XCR1 is selectively expressed on a conventional dendritic cell subset, the cDC1 subset, through phylogenetically distant species. The outcome of antigen-targeting to XCR1 may therefore be similar across species, permitting the translation of results from experimental models to human and veterinary applications. Here we evaluated in pigs the immunogenicity of bivalent protein structures made of XCL1 fused to the external portion of the influenza virus M2 proton pump, which is conserved through strains and a candidate for universal influenza vaccines. Pigs represent a relevant target of such universal vaccines as pigs can be infected by swine, human and avian strains. We found that cDC1 were the only cell type labeled by XCR1-targeted mCherry upon intradermal injection in pig skin. XCR1-targeted M2e induced higher IgG responses in seronegative and seropositive pigs as compared to non-targeted M2e. The IgG response was less significantly enhanced by CpG than by XCR1 targeting, and CpG did not further increase the response elicited by XCR1 targeting. Monophosphoryl lipid A with neutral liposomes did not have significant effect. Thus altogether M2e-targeting to XCR1 shows promises for a trans-species universal influenza vaccine strategy, possibly avoiding the use of classical adjuvants.

## Introduction

Targeting antigens to endocytic receptors expressed on dendritic cells is a potent way to enhance the efficiency of protein and DNA-based vaccines. To date, over a hundred preclinical studies have demonstrated that DC-targeting is efficient in many murine models to improve responses against model antigens such as ovalbumin, as well as against pathogen and tumor antigens, resulting in enhanced protection against infection and tumor challenges (for reviews see refs 1–4). However the translation of DC-targeting to other species such as livestock and primates has led to less spectacular outcomes^1, 5^. For instance, whereas over 20 different mouse studies show that DEC205 targeting stimulates high antibody responses, Th1 orientation and strong CD8^+^ T cell responses^4^, DEC205 targeting only induced low level CTL responses in non-human primates and did not improve the Th1 nor the antibody responses^6^. Thus it remains to be established whether DC-targeting has any advantage above classical adjuvants to ameliorate vaccination in application species.

The limited benefit of the DC-targeting in non-murine species may be due to the targeted immune receptor which, depending on species, can be expressed on non-DC type cells and may trigger undesired or counteractive responses. Furthermore the targeted molecules are most often differentially expressed by different subtypes of mononuclear phagocytes, with different patterns across species. The subtypes of mononuclear phagocytes include monocytes and monocyte-derived cells, tissue macrophages, Langerhans cells and the bona fide DC which encompass plasmacytoid DC (pDC) and the conventional DC (cDC) types cDC1 and cDC2 (see recent review^7^). The subtypes of mononuclear phagocytes have distinct functional attributes, with cDC subsets being the most specialized subsets in antigen presentation. Thus cDC are the most attractive cell types for antigen-targeting. Furthermore, whereas the cDC1 type excels in cross-presentation of antigens for CD8^+^ T cell priming, CD8^+^ T resident memory generation^8^ and recall responses^9^, they also are effective in T helper follicular activation for high antibody production^10^. Indeed the targeting of CLEC9A, a cell surface molecule highly expressed on murine cDC1 over on other DC types, drove a highly efficient development of follicular helper T cells^11^ and promoted affinity maturation of immunoglobulins in germinal centers in mice^10^. Furthermore, the targeting of influenza hemagglutinin to murine XCR1, the XCL1 receptor which is selectively expressed on cDC1^12, 13^, potently enhanced the CD8^+^ T cell response^14–16^ as well as the antibody response, especially of the IgG2a isotype^14^. Importantly we previously showed that XCR1 stands as a unique molecule selectively expressed by cDC1 across warm blooded species, i.e. mice, human, sheep and swine^17^. To the best of our knowledge, no other surface receptor selectively expressed on a DC subset is as widely conserved.

In this study, we assessed the efficiency of XCR1 targeting with a protein-based vaccine in pigs, which is an economically important livestock species and a biomedical model for the study of several human physiological and pathological conditions, including zoonotic infectious diseases, such as influenza infections. We selected the extracellular domain of the influenza A M2 proton pump (M2e), which is conserved across influenza strains and is a candidate B cell antigen for universal influenza vaccine development. M2e-specific Abs are responsible for the protective immunity induced by M2e vaccination: indeed the transfer of serum from M2e-immunized animals to naive recipients effectively protects against homologous and even heterologous challenges^18–20^. Anti-M2e IgG usually do not show viral neutralizing activity *in vitro* but Fc receptor-dependent mechanisms were shown to be critical in the protection, particularly through macrophages^19, 21^. As M2 is expressed at low levels on the virus particles but is abundant on infected cells^22^, anti-M2e IgG probably provide protection by interacting with virus-infected cells via antibody dependent cell-mediated cytotoxicity or phagocytosis. The role of complement appears negligible^19, 23^. However as M2e is a very weak and poorly accessible antigen in influenza particles, it needs to be properly exposed to induce protective immunity. We expressed 3 tandem repeats of M2e in fusion with porcine XCL1 (poXCL1-M2e) in bivalent structures named vaccibodies (VB) and delivered them intradermally in pigs, alone or in combination with monophosphoryl lipid A (MPL), a non toxic derivative of *Salmonella minnesota* LPS and a Toll-like receptor (TLR) 4 ligand, or CpG, a TLR9 ligand. VB contain the hinge and CH3 domain of human IgG3 to mimic the bivalent receptor binding capacity of an antibody and they incorporate a targeting unit (here poXCL1) and an antigenic part (here M2e) and yet remain smaller than an Ig molecule. We found that when injected intradermally, poXCL1-M2e VB induced higher anti-M2e IgG responses than non-targeted VB, and revealed to be more potent than TLR ligand adjuvants such as CpG and MPL administered with neutral liposomes.

## Materials and Methods

### Adjuvants and formulations

Squalene-in-water emulsion (SWE) was prepared as previously described^24^. Liposomes were manufactured by a lipid film-rehydration method followed by extrusion. The neutral liposomes (NL) are composed of 2.5 mg/mL cholesterol and 10 mg/mL of 1,2-dioleoyl-sn-glycero-3-phosphocholine [DOPC] in PBS pH 7.2, while the cationic liposomes (CL) are composed of 2.5 mg/mL cholesterol and 10 mg/mL of 1,2-dioleoyl-3-trimethylammonium-propane [DOTAP] in 20 mM Hepes and Glucose 5%. Monophosphoryl Lipid A (MPL) from *Salmonella enterica* serotype *Minnesota* was suspended in water for injection at 1 mg/mL, and ultra-sonicated with a probe sonicator and filtered (0.20 μm). This solution was added to the liposome suspensions in a 1:1 v/v ratio. Lipids were acquired from Avanti lipids and MPL from Sigma. CpG oligo-dinucleotides 5′-ggTGCATCGATTTATCGATTATCGATGCAGggggg-3′ with lower case letters for phosphorothioate linkages and upper case letters for phosphodiester linkages previously shown to be efficient in pigs^25^ were bought from Sigma.

### Vaccibody (VB) production

The generation of poXCL1-mCherry and anti-4-hydroxy-3-iodo-5-nitrophenylacetic acid (NIP) -mCherry VB has been described previously^26^. For generation of poXCL1-M2e and NIP-M2e, three consecutive copies of the M2e (3M2e) antigen were cloned into the poXCL1 and NIP VB backbones. The aa sequence of M2e (SLLTEVETPTRSEWESRSSDSSDAAASLLTEVETPTRSEWESRSSDSSDAAASLLTEVETPTRSEWESRSSDSSDAAA) was modified by replacing cysteines 17 and 19 of the original sequence with serine residues (underlined), to improve immunogenicity and avoid unwanted disulfide bounds in the chimera^27, 28^. The M2e sequence was derived from the sequence of the human A/PARIS/2590/2009 (H1N1) pandemic virus. poXCL1-M2e and NIP-M2e were produced by transient transfection of HEK293E cells in 5 layer flasks (BD Multi-Flask) using Lipofectamine 2000 (Life technologies). VB were purified by harvesting supernatants and applying them onto a Sepharose 4 Fast flow column (GE Healthcare) conjugated with the antibody HP6017, that bind the human CH3 dimerization domain. Bound VB were washed with PBS before elution with 0.1 M Glycin-HCl pH 2.7. Eluted protein were dialyzed in PBS and concentrated to 1 mg/ml using a 10 kDa cutoff Vivaspin column (Sartorius Stedim Biotech). Purified VB were subsequently stored at −80 °C until use. The concentration of the purified poXCL1-M2e and NIP-M2e VB were initially determined by a BCA protein assay kit. Correct size of the purified VB were determined by SDS-PAGE on a 10% Bolt^TM^ Bis-Tris Plus gel in MOPS Bolt^TM^ SDS Running Buffer (Life Technologies). In addition, normalized poXCL1-M2e and NIP-M2e were evaluated for equal quantities by ELISA using Costar 96-well plates coated with anti-human CH_3_ (MCA878, AbD Serotec). After blocking plates with 1% (w/v) BSA in PBS with 0.02% (w/v) Na Azide, normalized concentrations of VB were added and 3-fold serial diluted and incubated overnight at 4 °C. The plates were next washed and incubated with HP-6017-biotin (Sigma) diluted 1:1000 in PBS with 0.1% (w/v) BSA and 0.02% (w/v) Na Azide for 2 h at RT. After washing, streptavidin-ALP (Sigma) diluted 1:3000 in PBS with 0.1% (w/v) BSA and 0.02% (w/v) Na Azide was added and plates incubated 1 h at RT. The plates were subsequently washed and developed by adding 1 mg/ml phosphatase substrate (Sigma) dissolved in substrate buffer (9.7% w/v diethanol amine, 10.1% w/v MgClx6H_2_O and 0.02 (w/v) Na Azide in H_2_O). OD_405_ was measured using a TECAN microplate reader. Finally right before vaccination, the quality and quantity of the frozen stocks of the NIP and poXCL1-M2e were checked by the protein signals obtained in Coomassie blue staining of SDS-PAGE gel, under reducing conditions, relatively to a bovine serum albumin calibration, using the ChemiDc MP system (Bio-Rad).

### Animals

The animal experiments were approved by the French ethical committee #19, i.e. the Comité d’Éthique en Expérimentation Animale Val de Loire, under the number 00783.02 and comply to the French law on animal experimentation 2013-118 and to the directive 2010/63/EU. The animal experiments were done at the Plateforme d’Infectiologie Expérimentale PFIE-INRA, Nouzilly, France, under the accreditation number for animal experimentation C37-175-3 under A-BSL1 containment. Large-white pigs were obtained from the INRA conventional breeding unit Unité Expérimentale de Physiologie Animale de l’Orfrasière PAO-INRA, Nouzilly, France.

### Immunization experiments

In the case of experiment 1, 48 pigs (3–4 months of age) were randomly assigned into 8 groups of 6 pigs each (half males, half females in each group) originating from different mothers. The VB were delivered intradermally in a 450 µl volume in 4 spots (110 µl each) using hypodermic needle on day 0 (D0) and D28 under anesthesia (2 mg/kg xylazine and 10 mg/kg ketamine, intramuscular route), and the pigs were culled on D56. Four groups received 0.25 µg VB and 4 groups received 2.5 µg VB per pig either in plain PBS or formulated with 100 µg MPL+ neutral liposomes (NL). Serum was collected on D0, 28 and 56. Two pigs were prematurely culled due to lameness not resolved with anti-inflammatory drug treatment. In the case of experiment 2, 32 pigs (2 months of age), originating from 8 different mothers were serologically tested for anti-H1N1 and anti-NP IgG levels 2 weeks before the first vaccination. All mothers were seropositive for influenza and presented similar anti-H1N1 and anti-NP IgG levels. The piglets were assigned to 4 groups of 8 piglets, randomized based on anti-H1N1 and anti-NP IgG levels, mother of origin, and sex. The influenza seropositivity was due to previous exposure of the mothers or of the piglets to an influenza virus, not to vaccination.

The VB (8 µg) were delivered intradermally in a 450 µl volume using hypodermic syringe on D0, D28 and D55 under anesthesia, with or without 500 µg CpG, and the pigs were culled on D85. Serum was collected on D0, D55. D85. Five pigs were culled prematurely due to rectal prolapsus.

### Skin cell targeting by VB

NIP-mCherry and poXCL1-mCherry VB (60 µg each) were mixed with different formulations in PBS except in the case of cationic liposomes (CL) where a 5% glucose +20 mM Hepes buffer was used, in a final 600 µl volume. The volume ratios were 50:50 for the VB:SWE adjuvant, 60:20:20 for the VB:NL:MPL (100 µg), and 60:20:20 for the VB:CL:MPL (100 µg). The formulated VB were injected intradermally in 8 spots (75 µl each) using an hypodermic needle under general anesthesia. One pig was used for each formulation for which the NIP-mCherry and the poXCL1-mCherry VB were injected in the left and in the right inguinal area respectively. After 24 hours, the pigs were culled and the injected skin spots were collected and placed on ice. After removal of fat, the injected skin spots were cut in 4, pooled and digested overnight in RPMI +10% FCS + collagenase D (1 mg/ml, Roche) + dispase (0.5 mg/ml, InVitrogen) + antimycotic antibiotic cocktail. The cells were then filtered through 100-μm pores-nylon mesh filters. For staining, 10^6^ cells per tube were incubated for 30 min on ice in blocking buffer (PBS + 5% horse serum + 5% pig serum) and stained with anti-MHC class II mAb (MSA3 clone, IgG2a, Washington State University Monoclonal Antibody Center, Pullman, Wa (WSU)) and anti-CD172 mAb (clone 74-22-15, WSU) at a 2 µg/ml concentration followed by Alexa 488 and Alexa 647 - conjugated goat IgG anti-mouse IgG2a and IgG1 respectively. Dead cells were excluded with DAPI. The isotype IgG2a and IgG1 control was done on a pool of skin cells injected with the different formulations. Flow cytometry acquisition was done with a Becton Dickinson Fortessa Flow cytometer and the acquired data were analyzed using FlowJo software (version X.0.6, Tree Star).

### Ab ELISA

Individual pig sera were assayed for anti-M2e-specific IgG by ELISA at D0 and on the indicated days. Fifteen days before experiment 2, piglets and their mothers’ sera were tested for anti-NP and H1N1-specific IgG. Half area flat bottom polystyrene high bind microplates (Corning) were coated overnight at 4 °C with 100 ng M2e peptide or 200 ng NP (described in ref. 29) or 200 ng inactivated A/PARIS/2590/2009 H1N1 in 50 μl of PBS. Plates were saturated with 5% FCS in PBS −0.05% Tween 20 for 1 h at 37 °C. Samples were 2-fold serially diluted starting at 1:30 and were incubated for 2 h at 37 °C. Antigen-bound IgG were detected using horseradish peroxidase (HRP)-conjugated goat anti-pig IgG Fc fragment (Bethyl Laboratories, Montgomery, USA) at 10 ng/ml. Antigen-bound pig IgG1 and IgG2 were detected with murine mAb anti-pig IgG1 (clone K139 3C8, IgG1 isotype, Bio-Rad) at 10 µg/ml and murine mAb anti-pig IgG2 (clone K68-Ig2, IgG1 isotype, Bio-Rad) at 1 µg/ml followed by HRP-conjugated rat anti-mouse IgG1 at 500 ng/ml (BD Biosciences). All detection antibodies were incubated for 1 h at 37 °C. The ULTRA-tetramethylbenzidine HRP substrate (TMB, Thermo-Scientific) was used and absorbance was measured at 405 nm and the results were expressed as endpoint antibody titers, calculated by regression analysis plotting dilution versus *A*_405_. Endpoint titers were calculated as the highest dilution giving twice the absorbance of the negative control.

### Isolation of splenocytes and depletion of γ/δ and CD8^+^ or CD4^+^ T cells

Spleen cells were isolated by mechanical dissociation followed by filtration on successive 500 µm and 100 µm mesh-sized nylon filters and centrifugation on Ficoll-Hypaque density gradient. Spleen cells were step frozen in FCS +10% dimethyl sulfoxide and stored in liquid nitrogen. Depletion of γ**/**δ and CD8^+^ T or of γ**/**δ and CD4^+^ T cells was performed with indirect magnetic sorting according to the manufacturer’s instructions (Miltenyi Biotec). γ/δ T cells were removed as these cells have been shown to display regulatory properties in cattle, another artiodactyl species^30^. In brief, splenocytes were incubated with 0.5 µg/10^7^ cells of anti-porcine γ**/**δ T cell receptor (mix of 86D and PGL22A clones (Washington State University (WSU), USA)) and together either with anti-porcine CD8β (PG164A, WSU) or with anti-porcine CD4 (74-12-4, WSU) and followed by incubation with 20 µl goat anti-mouse IgG microbeads and depletion using LD columns (Miltenyi Biotec). Depletion was checked by staining with anti-mouse IgG conjugated secondary antibody in flow cytometry and was found to be less than 1%.

### T-cell restimulation and ELISPOT

The number of antigen-specific IFNγ-secreting splenocytes were determined using the porcine IFNγ ELISPOT assay, following the recommendations from MabTech AB (Nacka Strand, Sweden). MultiScreen HTS plates (Millipore) were pre-coated overnight with 15 µg/ml capture anti-pig IFNγ mAb in PBS. Spleen cells were thawed from frozen stocks and dead cells were removed with an optiprep gradient. Cells were resuspended in X-vivo medium (Ozyme, Saint-Quentin-en-Yvelines, France) supplemented with 2% FCS and penicillin/streptomycin and 2.5 × 10^5^ were plated in the pre-coated ELISPOT plate and stimulated with 5 µg/ml overlapping peptides covering the CH3 and hinge region of human IgG3 and M2e sequences of the vaccibodies (20 mers, offset 8, Mimotopes Pty Ltd, Victoria, Australia). Unstimulated wells included 5 µl of H20: acetonitril peptide diluent (vol:vol). A peptide derived from the HIV polymerase was used as negative control (5 µg/ml) and ConA (25 µg/ml) was used as positive control. After 18 h, the IFNγ-secreting cells were revealed by sequential incubations with 0.5 µg/ml biotinylated anti-IFNγ followed by 0.5 µg/ml alkaline phosphatase conjugated-streptavidin and BCIP/NBT reagent. The number of specific IFN-γ secreting cells was determined by the AID ELISPOT reader.

### Statistical analyses

Data were analyzed with the GraphPad Prism 5.0 software. Unpaired t-tests were used for comparison of antibody responses between 2 vaccinated groups, bilateral in experiment 1, and monolateral in experiment 2.

### Data availability

Materials, data and associated protocols are available to readers upon request to the corresponding author.

## Results

### poXCL1-mCherry selective interaction with skin cDC1 *in vivo* depends on the formulation used for adjuvant combination

**Figure 1:**
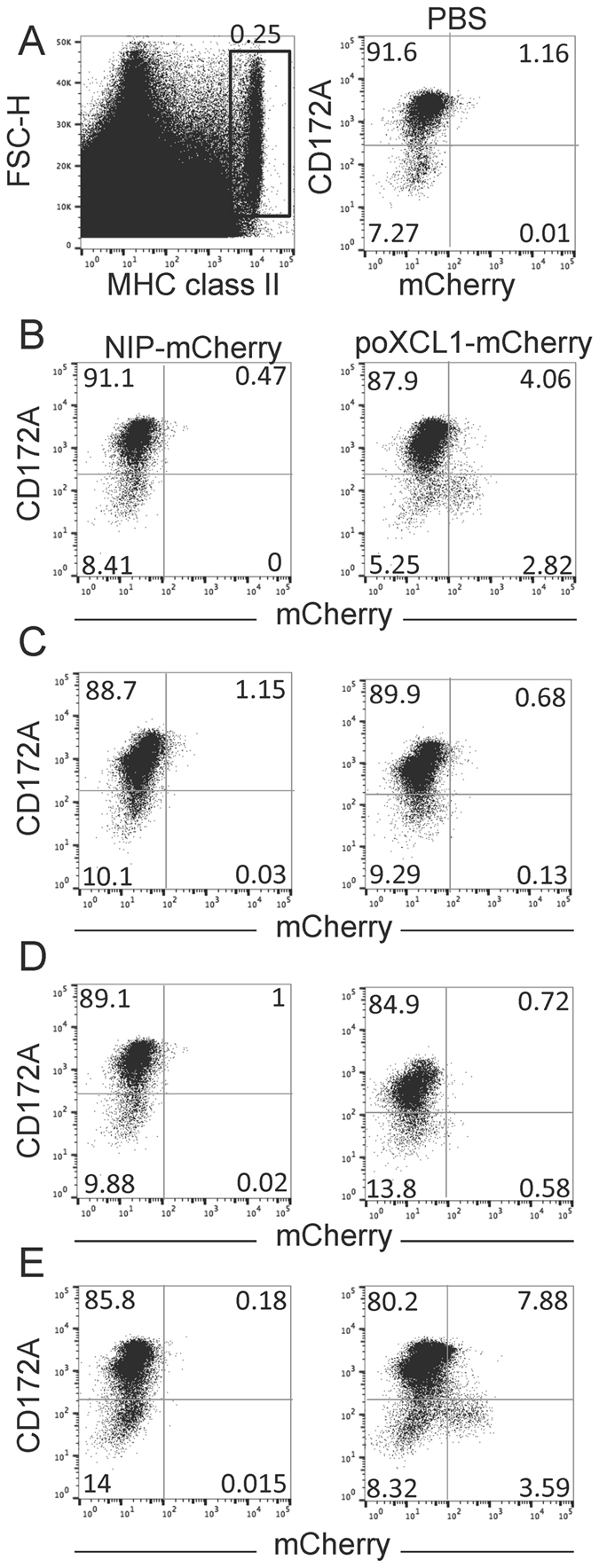
Selective targeting of pig skin cDC1 subset with poXCL1-mCherry upon intradermal injection with different adjuvant formulations. NIP-mCherry and poXCL1-mCherry (60 µg) were injected intradermally in different adjuvant formulations, skin cells were collected after 24 h by enzymatic dissociation and labeled for MHC class II and CD172A detection. FSC^high^ and MHC class II^high^ cells (cDC) were gated as shown in A (left panel) and their percentage among total extracted skin cells is indicated. The FSC^high^ and MHC class II^high^ gated cells were analyzed for CD172A and mCherry expression (in A (right panel) to E) and the percentage of each quadrant among gated cells is indicated. The injections are: in A (right panel), PBS; in B, plain VB; in C, VB formulated with SWE adjuvant; in D, VB formulated with CL + MPL; in E, VB formulated with NL + MPL.

We recently published that vaccibodies (VB) made of pig XCL1 in fusion with mCherry (poXCL1-mCherry) selectively interacted with pig blood cDC1 as well as skin cDC1 from culture explants *in vitro* ^26^. These observations suggest that poXCL1-fusion vaccines can be used to immunize pigs. However, to ensure efficient targeting of cDC1 *in vivo*, poXCL1-mCherry was delivered into the skin by intradermal injection and the injection site was excised and enzymatically digested after 24 h. As shows Fig. 1A (left panel), the FSC^hi^ MHC class II^hi^ cells, which correspond to pig skin DC^31^, represent about 0.25% of the total skin cells. CD172A^−^ FSC^hi^ MHC class II^hi^ cells correspond to cDC1 in pig skin^31^. In this experiment, poXCL1-mCherry selectively clearly labeled 35% of the CD172A^−^ FSC^hi^ MHC class II^hi^ cells of enzymatically-extracted skin cells (2.82% of total DC), as compared to the NIP-mCherry negative control (VB containing a scFv specific for the hapten NIP, Fig. 1B). The labeling with poXCL1-mCherry may correspond to internalized, membrane-bound forms or both. Some CD172A^+^ cells were very faintly labeled with poXCL1-mCherry, possibly related to the chemokine interaction with surface glycosaminoglycans^32^. There was no mCherry signal in DC from the NIP-mCherry VB injections when compared to PBS (Fig. 1A, right panel), indicating very limited non-specific binding of NIP-mCherry and/or rapid lysosomal degradation following non-specific receptor-mediated pinocytosis^33^. Finally the fraction of non-labeled cDC1 with poXCL1-mCherry might be explained either by lack of access of the inoculum to all the cDC1 present in the skin at the time of the digestion or possibly by the degradation/quenching of the poXCL1-mCherry which is likely to occur with time upon internalization. As addition of adjuvants has been shown to be beneficial and sometimes essential to the immunogenicity of DC-targeted antigens in the mouse model^34–36^, we evaluated the interaction of poXCL1 and NIP-mCherry to cDC1 when mixed with squalene-in-water emulsion (SWE adjuvant), with MPL + cationic liposomes (CL) or with MPL + neutral liposomes (NL) injected intradermally. We selected these adjuvants because vaccines including squalene and MPL adjuvants have been licensed for human use. CL or NL were added to MPL with the goal to improve its bio-availability and to potentiate the immunostimulatory effect^37^. As shown Fig. 1C and D, SWE and CL formulations prevented the interaction of poXCL1-mCherry to pig skin cDC1, and in addition induced a strong local inflammation 24 h–96 h post injection at the difference with plain VB (Supplementary Fig. 1A,B,C). Interestingly, MPL + NL formulation maintained poXCL1-mCherry targeting to 30% of the cDC1 (Fig. 1E, 3.59% of the total DC) and was minimally inflammatory (Supplementary Fig. 1D). We thus selected MPL + NL for the experiment 1 of XCR1-targeting in pigs using the VB strategy.

### Production and purification of poXCL1-M2e and NIP-M2e VB

**Figure 2:**
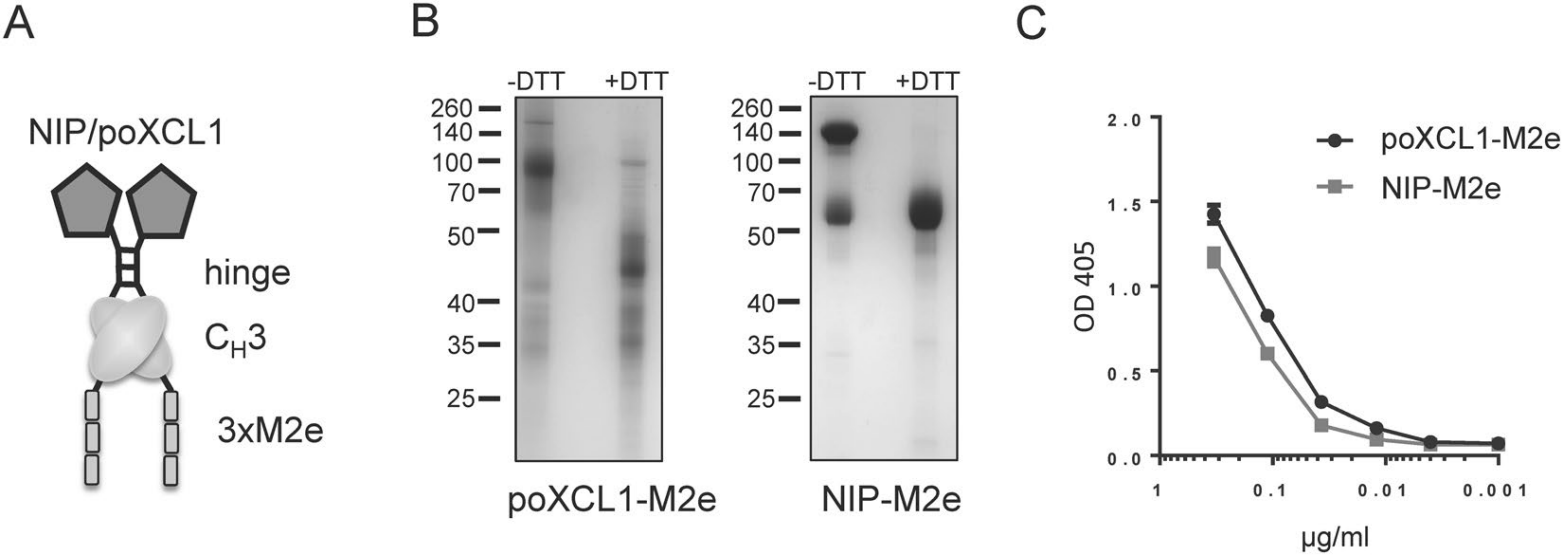
Characterization of poXCL1-M2e and NIP-M2e VB. In (**A**) the M2e VB consist of a targeting unit (poXCL1 or NIP), a dimerization domain with hinge and CH_3_ from human IgG3 and an antigenic unit with three repeats of the M2e sequence. In (**B**) purified poXCL1-M2e (predicted size 33 kDa) and NIP-M2e (predicted size 48 kDa) were analyzed by SDS-PAGE under reducing (+DTT) and non-reducing (-DTT) conditions. In (**C**) normalized concentrations of purified poXCL1-M2e and NIP-M2e preparations were evaluated by ELISA, and detected using an antibody specific for the VB dimerization domain.

Influenza M2e was selected as the antigen to be included in VB for targeting pig XCR1, as M2e represents an evolutionary conserved influenza antigen which is promising for generating universal influenza vaccines. Although M2e is poorly immunogenic in its natural context, it has been shown to induce protective immunity in mouse and ferret models when tandem repeats were expressed in fusion with different types of carriers^27, 38^. Consequently, we fused three tandem repeats of M2e from pandemic H1N1 virus at the C terminal end of the CH3 domain of VB containing NIP or poXCL1 as targeting units at the N terminal end (Fig. 2A). The NIP portion corresponds to an irrelevant scFv for making VB with untargeted M2e. poXCL1-M2e and NIP-M2e were expressed and purified as previously published^14^. The purified VB proteins were evaluated for size and dimerization by SDS-PAGE (Fig. 2B). For NIP-M2e VB, we observed the presence of monomeric VB in the non-reduced samples, although the strongest band correlated with the dimeric molecule. For poXCL1-M2e VB, the main band correlated to the dimeric molecule. We did however observe a more smeary background which is similar to previous observations with poXCL1-mCherry^26^. The concentration of the purified poXCL1-M2e and NIP-M2e VB were initially determined by BCA protein assay kit. In addition, to ensure equal amounts of VB in the two normalized protein preparations, an ELISA was performed where we detected the dimerization domain of the VB (Fig. 2C).

### XCR1 targeting with VB in skin enhances M2e immunogenicity in seronegative pigs

**Figure 3:**
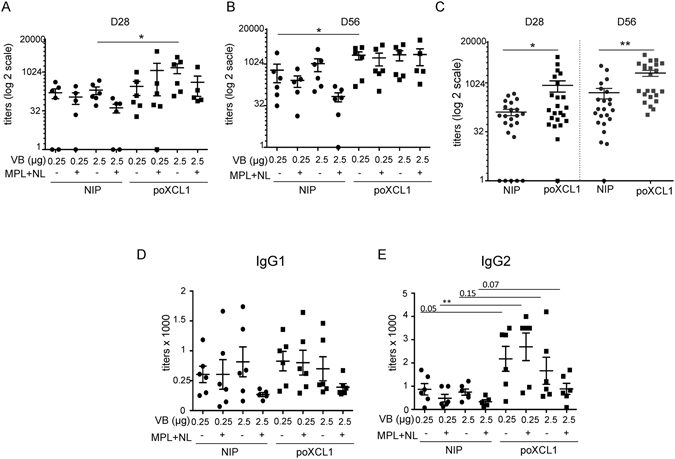
Effect of XCR1 targeting and MPL adjuvant on the anti-M2e IgG response in seronegative pigs. Pigs were inoculated twice (D0 and D28) intradermally with 0.25 or 2.5 µg of poXCL1-M2e or NIP-M2e VB with or without CL + MPL. In (**A** and **B**), the anti-M2e IgG titers obtained in the different groups at D28 (**A**) and at D56 (**B**) are shown. Signals with sera from D0 were all at the level of background. In (**C**), as neither the dose nor the formulated MPL were confounding factors, the NIP-M2e and the poXCL1-M2e vaccinated groups were combined to increase the statistical power. In (**D** and **E**), the IgG1 and IgG2 anti-M2e titers at D56 are shown for the different groups. In all panels, each dot corresponds to individual pig values. Arithmetic means and standard errors of the mean are shown. Statistical significance between 2 groups was calculated with unpaired t-test (p values are reported when > 0.05, * < 0.05; ** < 0.01).

poXCL1-M2e and NIP-M2e (0.25 or 2.5 µg, 6 pigs per groups, experiment 1) were inoculated intradermally into influenza seronegative piglets, in saline or in MPL + NL, twice at 28 days interval (D0 and D28). Serum samples were harvested at day 28 before the boost, and at day 56 and evaluated for the presence of M2e specific IgG. A tendency for higher IgG responses was obtained in the groups injected with poXCL1-M2e compared to NIP-M2e (Fig. 3A and B, p < 0.05 for the 2.5 µg condition without MPL + NL at D28, p < 0.05 for the 0.25 µg condition without MPL + NL at D56). No effect of MPL + NL addition nor of VB dose were found at both time points (Fig. 3A and B). In order to increase the statistical power, we compared the IgG titers in the sera of all the poXCL1-M2e versus the NIP-M2e inoculated pigs (Fig. 3C), as the doses and formulations were not confounding factors. After one injection, 6 sera out of 23 with titers above 1000 were found in the poXCL1-M2e and none in the NIP-M2e injected groups, only one pig out of 23 in the poXCL1-M2e and 5 pigs out of 23 in the NIP-M2e groups were not responding, and the IgG titers were superior in the poXCL1-M2e groups (p < 0.05). After 2 injections, 11 sera out of 23 with titers above 1000 were found in the poXCL1-M2e and 4 sera out of 23 in the NIP-M2e injected groups and the IgG titers were superior in the poXCL1-M2e groups (p < 0.01). Finally, commercial reagents exist to differentiate pig IgG1 and IgG2, which are distinct swine IgG isoforms separated by ion exchange chromatography^39^. Neither MPL + NL nor XCR1 targeting affected the IgG1 levels (Fig. 3D), however the IgG2 levels were enhanced by XCR1 targeting (Fig. 3E).

### XCR1 targeting with VB in skin enhances M2e immunogenicity in seropositive pigs

**Figure 4:**
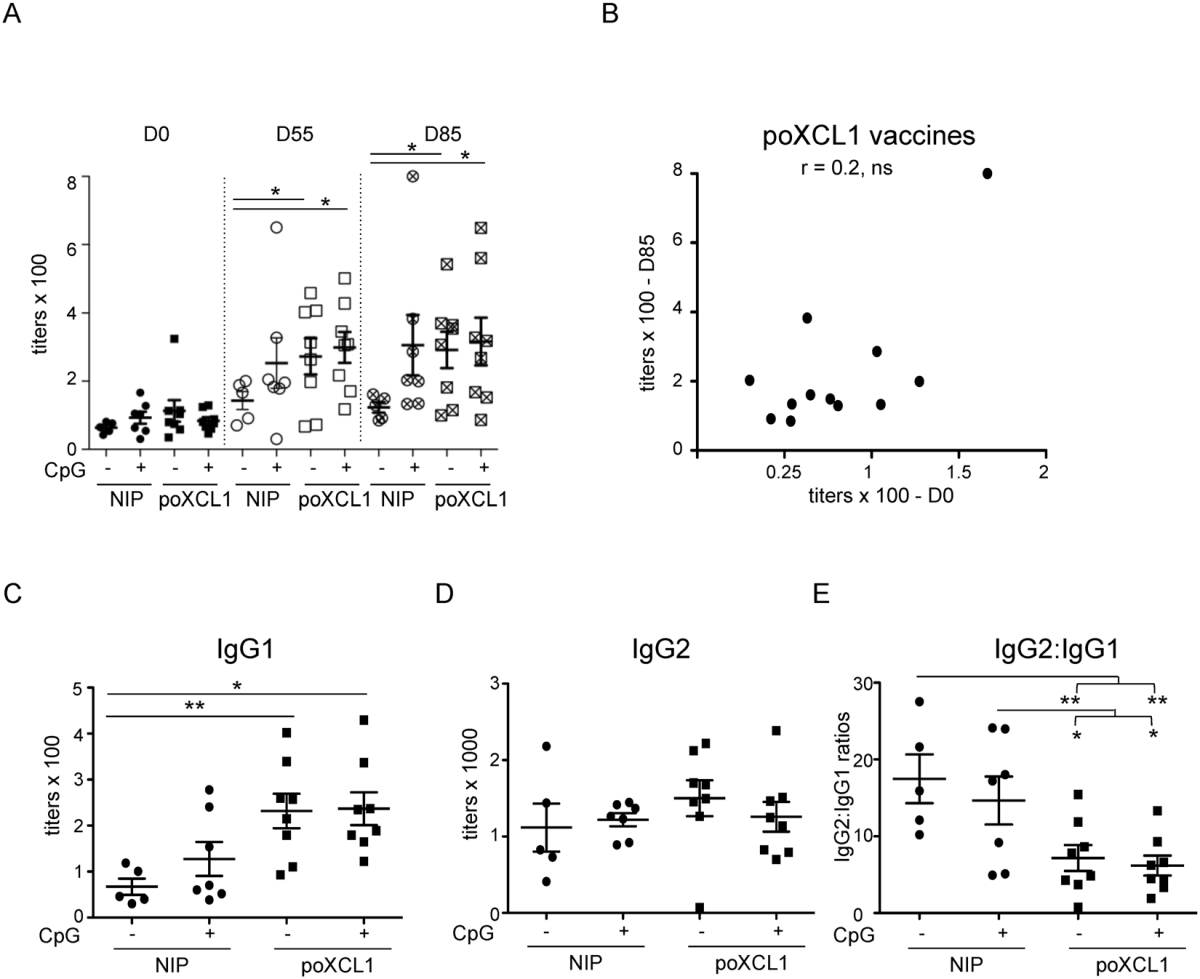
Effect of XCR1 targeting and CpG adjuvant on the anti-M2e IgG response in seropositive pigs. Pigs with pre-existing antibodies against M2e were inoculated 3 times intradermally (D0, 28, 55) with 8 µg poXCL1-M2e or NIP-M2e VB with or without CpG. In (**A**), the anti-M2e IgG titers obtained in the different groups at D0, D55 and D85 are shown. In (**B**), the D0 versus D85 anti-M2e IgG titers from poXCL1-M2e vaccinated pigs (with and without CpG) were plotted and correlation was estimated with a two-tailed Pearson test. In (**C** and **D**), the IgG1 and IgG2 anti-M2e titers at D85 are shown for the different groups. In (**E**) the IgG2 titer: IgG1 titer ratios are depicted. In all panels, each dot corresponds to individual pig values. Arithmetic means and standard errors of the mean are shown. Statistical significance between 2 groups was calculated with unpaired t-test (p values = * < 0.05; ** < 0.01).

As universal influenza vaccines are expected to be administered into individuals with pre-existing immunity against influenza, we immunized piglets presenting significant levels of anti-M2e IgG at D0, using 8 µg VB in 3 injections (at D0, 28 and 55, Experiment 2, Fig. 4A). In this case, since MPL + NL did not improve the vaccination efficacy, we used a pig CpG as an adjuvant. The pig CpG sequence used in this study contains the palindromic hexamer ATCGAT which was identified to be optimal for cytokine production by pig pDC^40, 41^ as well as for pig B cell activation^42^ and it has been demonstrated to improve the immune responses in pigs *in vivo* ^25^. The anti-M2e IgG titers were generally lower than in experiment 1. The group vaccinated with poXCL1-M2e with and without CpG showed higher anti-M2e IgG titers than the group vaccinated with NIP-M2e alone at D55 and D85, (Fig. 4A, p < 0.05). There was no clear differences between the titers measured at D85 versus D55, indicating that the third boost did not have much effect. CpG had a slight positive effect on the anti-M2e levels in the NIP-M2e vaccinated pigs, although the difference was not statistically significant, and CpG did not potentiate the anti-M2e titers seen with poXCL1-M2e. The level of pre-existing antibodies did not seem to compromise the anti-M2e responses induced by the poXCL1-M2e, as there was no negative correlation between pre-existing anti-M2e IgG and anti-M2e IgG at D85 (Fig. 4B). We further tested whether the VB structure (hinge, CH3 and M2e fusion) could have served as T cell antigens for eliciting CD4^+^ or CD8^+^ T responses. However, very scarce to no IFNγ-secreting cells were detected among total, CD4^+^ or CD8^+^ T cell-enriched splenic populations restimulated with overlapping peptides covering the VB structure (below 10 cells per 2.5 10^5^ cells, Supplementary Fig. 2). While poXCL1-M2e preferentially induced IgG2 in seronegative pigs (Fig. 3), XCR1 targeting enhanced IgG1 titers, but not IgG2 titers, in the seropositive pigs, irrespective of the use of CpG (Fig. 4C and D). The anti-M2e IgG1 titers measured at D0 were slightly lower than the IgG2 titers (see Supplementary Fig. 3). Thus the anti-M2e IgG types of the pre-existing immunity, either resulting from infection or maternal antibodies, were slightly biased towards IgG2 (mean ratio IgG2:IgG1 titers = 1.96 ± 0.89). In that context, the bias was much increased by immunization with NIP-M2e (mean ratio IgG2:IgG1 titers = 17.48 ± 5.6 and 14.23 ± 7.06 with and without CpG respectively, Fig. 4E) and less by immunization with poXCL1-M2e VB (mean ratio IgG2:IgG1 titers = 6.77 ± 3.76 and 6.45 ± 2.67 with and without CpG respectively, Fig. 4E).

Altogether the results show that M2e-targeting to XCR1 promotes the IgG response against M2e in pigs, to a higher extent than CpG and MPL.

## Discussion

In this study, we showed that targeting the evolutionary conserved M2e influenza antigen to XCR1 using VB platforms promotes anti-M2e antibody responses in pigs, and thus stands as a promising approach towards the development of a universal vaccine against influenza. Addition of adjuvants, i.e. the TLR4 ligand MPL with NL or the TLR9 ligand CpG, did not further increase the antibody levels induced by the poXCL1-M2e VB.

The improvement of the anti-M2e IgG response induced by poXCL1-M2e VB was less marked in seropositive than in seronegative pigs. Indeed preexisting Ab to influenza can alter the immune response to vaccines^43, 44^. In our study, the pre-existing Ab could either result from passive immunity with maternal transfer or active immunity in case of exposure to the virus, though it is difficult to differentiate these two possibilities a posteriori. In any event, we did not observe negative correlations between the levels of pre-existing Ab and the vaccine-induced Ab, suggesting that targeting an antigen to XCR1 can enhance antibody responses in both naïve pigs and pigs with pre-existing immune responses. However, it is puzzling that poXCL1-M2e preferentially stimulated the production of anti-M2e IgG2 in seronegative pigs and of anti-M2e IgG1 in pigs which were seropositive at priming. These IgG1 and 2 forms correspond to the extremes of the ion exchange chromatography spectrum of IgG and should not be confused with mouse IgG1 and IgG2a subclasses^39, 45^. Indeed, diversification of IgG into subclasses appear to have occurred after speciation and the IgG1 in one species does not phylogenetically nor functionally correspond to IgG1 in another species^39^. As pig IgG1 and 2 are biochemically distinct, they might differ in function, although this needs to be investigated. In any event, our observations suggest that IgG1 and IgG2 productions in pigs can be oriented by the immunological priming context, such as the combination of pre-existing immunity and XCR1 targeting. In mice, the presence of maternal Abs has an influence on the Th1–Th2 balance in the offspring^46^ and XCR1 targeting preferentially orient Th1 responses^14^. The combination of the two factors has not been evaluated in this biologically relevant species.

The lack of detection of anti-M2e IgG in 5 pigs from the NIP-M2e vaccinated groups may be explained by their low capacity for VB antigen presentation in relation to their MHC haplotypes, a high activation threshold of their B cell receptors interacting with M2e epitopes and/or of their T cell receptors interacting with the VB peptides presented on their MHC molecules. The pigs used in this study, although not MHC typed, were likely to be heterogeneous at their MHC locus, given the breeding scheme used in the animal facility. XCL1-mCherry VB were shown in mice to remain at the cell membrane or be rapidly internalized upon interaction with XCR1, depending on the murine or human origin of XCL1^32^. Interestingly the persistence at the cell membrane was associated to higher capacity for antibody induction, possibly by increasing the duration of interaction with the BCR^32^. In addition, cDC1 excel in follicular T helper cell activation^11^. Whereas we do not know whether poXCL1-M2e is rapidly internalized or not, we can speculate that poXCL1-M2e interaction with pig cDC1 favors the crosstalk between cDC1, follicular T helper cells and B cells and thus bypass the threshold of BRC activation, even in the case of intrinsically poorly responding pigs to M2e antigen.

The enhancement of humoral responses with DC-targeted antigens in absence of additional adjuvants has been obtained in mice in several instances whereas the increase in T cell responses, and especially in CD8^+^ T cell responses, required the combination with adjuvants^47^. The enhancement of the humoral responses without adjuvant depended on the targeted receptors and was observed for some antigen-targeted receptors, such as CIRE and FIRE^48^ and CLEC9A^49^, but not in the case of DEC205^49^, DCIR2^50^ or Dectin 1^51^. In addition, the lack of requirement for adjuvants appeared to depend on helper peptides available in the Ig backbone of the antibody used for targeting, such as motives in the IgG2a backbone in the case of C57BL6 mice^49^. Similarly in non-human primates, an increase of antibody response independently of adjuvant has been observed in the case of antigen targeting to CLEC9A^49^, DCIR^52^, and langerin^53^. No further increase could be obtained with TLR7/8 ligand^53^ nor double stranded poly(I:C)^52^, suggesting that a maximal antibody response had been reached or that a key factor was limiting. In these cases, the Ig backbone used for targeting may also have played a role and may be involved in the heterogeneity of the responses across individuals. In the case of our VB, little to no T cell response against the hinge, CH3 and M2e portions could be detected. Thus we can speculate that much higher anti-M2e IgG levels would be obtained with our poXCL1-M2e strategy by including in the VB a strong and promiscuous T cell antigen such as tetanus toxoid in humans or the foot and mouth disease VP4 20–35 epitope in pigs^54^. The need for T cell epitopes in DEC205-targeting for improving antibody production has been recently documented^55^. Consequently we could possibly improve the VB for use in pigs by replacing the human sequences with pig sequences to avoid unwanted interference due to xenogeneic sequences and by including known strong and promiscuous T cell epitopes.

In this study, we tested additional adjuvants and formulations together with XCR1 targeting. We found that the oil-in-water emulsion (SWE) and the cationic liposome (CL) formulations induced some inflammation in our model and prevented the selective targeting of poXCL1-M2e to XCR1 on skin cDC1. In these instances, the VB could have been adsorbed on CL supposedly via electrostatic interactions, possibly captured by newly recruited inflammatory cells, and thus impeded in their capacity to selectively interact with XCR1 on skin cDC1. We selected a NL formulation for combination with MPL and VB as NL are a classical carrier to formulate MPL^37^, and NL by themselves have been shown to promote Th2 responses^56^, and NL did not modified VB interaction with cDC1 *in vivo*. However NL + MPL did not improve the antibody response induced by the NIP-M2e and the poXCL1-M2e in our setting. MPL has been investigated as a promising adjuvant in many preclinical studies and clinical trials and is included in an approved prophylactic human papilloma virus vaccine^37, 57^. To our knowledge, only one report mentions the use of MPL in pigs, where *Haemophilus ducreyi* hemoglobin receptor combined with MPL conferred protection against infectious challenge^58^. However, the comparison with the antigen alone was not evaluated in this study. A recent study conducted *in vitro* on pig DC subsets isolated from blood showed that LPS, from which MPL is derived, was less potent than other TLR ligands to stimulate cytokine production and activation markers’ expression in pig cDC1, cDC2 and pDC^59^. Furthermore TLR4 ligands show species preference profiles, with MPL being more efficient to stimulate mouse than human cells^60^, thus MPL may not be an optimal TLR4 ligand in swine. Besides, Auray *et al*. showed that TLR ligands and particularly TLR7 and 9 ligands, activate pDC and not cDC1 and cDC2 for the production of cytokines, despite expression of the TLR by the cDC^59^. Furthermore the up-regulation of the CD40 and CD86 molecules on cDC1 and cDC2 induced by the TLR ligands was dependent on the presence of pDC^59^. As pDC are virtually absent in skin at steady state, the intradermal delivery of MPL or CpG together with poXCL1-M2e may not have been able to activate pDC in the close vicinity of the targeted DC for achieving their optimal stimulation. Other adjuvants which directly activate cDC such as STING ligands and C-type lectin agonists^61^ and/or which would be co-delivered with VB to the same cDC remain to be evaluated to optimize the immunogenicity of VB when injected in the dermis in pigs. Finally TLR ligands might be more efficient if injected subcutaneously, a route which rapidly drains the antigens to lymph node where pDC are located and/or readily recruited^62^. Altogether, additional work is required to determine if XCR1 targeting of antigens can be or not further promoted by additional adjuvants and this will require testing suitable types of adjuvants, formulations and routes of administration.

Our chosen strategy here is to target antigens to a receptor which is selectively expressed by a specific DC subset. However, the targeting of molecules expressed concurrently by several antigen presenting cell types has also led to improved antibody responses in different species. This is the case of MHC class II targeting, as demonstrated in pigs^63^, in cows^64^ and recently by some of us in pigs and ferrets^65^. Indeed MHC class II-targeting may operate through promoting positive interactions between the targeted subsets, such as DC and B cells which express the molecule^66^. Interestingly, some of these studies revealed that the delivery of the antigen targeted to antigen-presenting cells in the form of DNA vaccines appeared to be highly efficient in pigs^63, 65^ and encourage the use of this delivery mode to further improve the efficacy of vaccines targeted to XCR1. XCR1 appears as a particularly efficient DC receptor for targeting vaccine antigen not only in the mouse model^14, 15, 32, 67^, but also in a large mammal pertinent to human, as we show here. In contrast, we published recently that the targeting of M2e to CD11c in the dermis did not improve the antibody response to M2e in swine^29^.

Altogether we report a promising strategy towards the development of universal influenza vaccine with the finding that M2e-targeting to XCR1 stimulated the anti-M2e IgG response in pigs, and revealed to be more efficient than MPL and CpG TLR ligands. This strategy could probably be further improved by adding T cell epitopes and by optimizing the delivery method such as with DNA vectors, for promoting the magnitude, the breadth and possibly the duration of the immune response, and possibly avoid the use of additional adjuvants. Future experiments should include challenge studies in pigs with heterologous viral strains to evaluate the cross-strain protective efficacy of M2e targeted to XCR1. The strategy could then be rapidly translated to species sensitive to influenza such as human, pigs, horses and even birds which also express XCR1 on cDC^68^.

## Electronic supplementary material

Supplementary information
