# Supplementary material for: The anti-influenza M2e antibody response is promoted by XCR1 targeting in pig skin
Source: Sci Rep. 2017 Aug 9;7:7639. doi: 10.1038/s41598-017-07372-9 (PMC5550447; doi:10.1038/s41598-017-07372-9)
Supplement: Supplementary file 1 — Supplementary information [file 41598_2017_7372_MOESM1_ESM.pdf]

Supplementary information

The anti-influenza M2e antibody response is promoted by XCR1 targeting in pig skin

Charlotte Deloizy, Even Fossum, Christophe Barnier-Quer, Céline Urien, Tiphany Chrun, Audrey Duval, Maelle Codjovi, Edwige Bouguyon, Pauline Maisonnasse, Pierre-Louis Hervé, Céline Barc, Olivier Boulesteix, Jérémy Pezant, Christophe Chevalier, Nicolas Collin, Marc Dalod, Bjarne Bogen, Nicolas Bertho, Isabelle Schwartz-Cornil

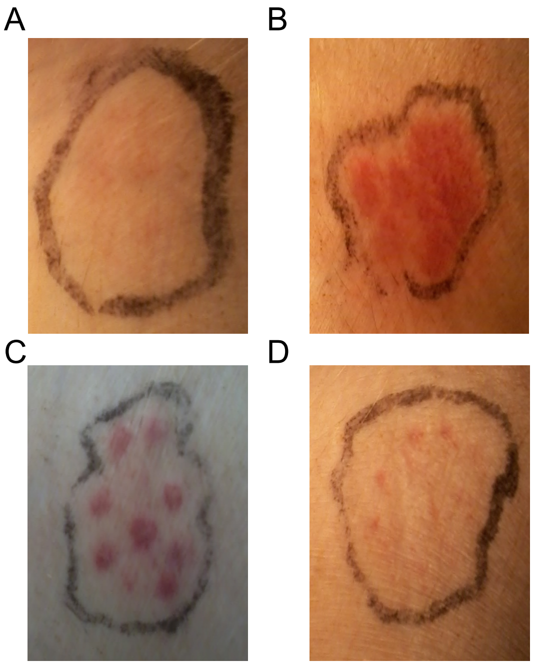

Supplementary Fig. 1. Skin reactivity to the intradermal inoculation of several formulations for the delivery of VB. NIP-mCherry and poXCL1-mCherry VB (60 µg) were injected intradermally in different formulations in 8 separated 75 µl spots and the skin reaction was examined at 24 H. The injected VB are: in A, plain VB; in B, VB formulated with SWE adjuvant; in C, VB formulated with CL + MPL; in D, VB formulated with NL + MPL.

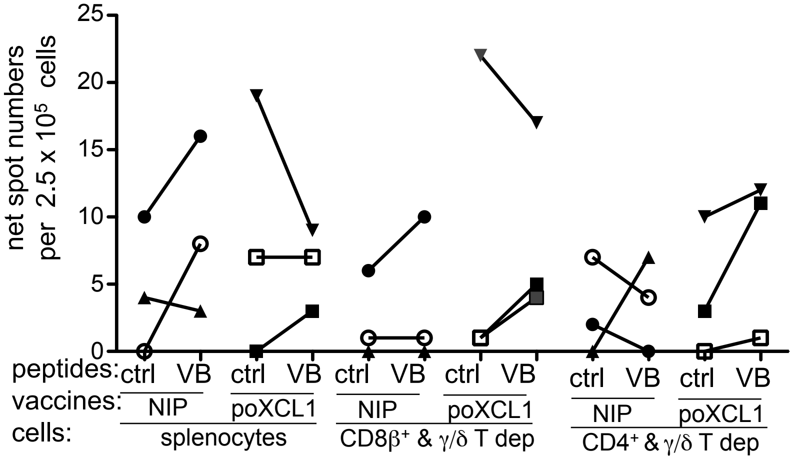

Supplementary figure 2. VB-specific IFNγ T cell responses of splenocytes from representative pigs immunized with NIP-M2e or poXCL1-M2e. Total splenocytes, as well as CD8β and γ/δ-depleted and CD4 and γ/δ-depleted splenocytes from three NIP-M2e and poXCL1-M2e immunized pigs (experiment 2) were restimulated with overlapping peptides from VB backbone corresponding to the CH3 and hinge of human IgG3 and M2e peptide (designated as VB, 5 µg/ml) or from a control peptide (ctrl, 5 µg/ml). ConA stimulation (25 µg/ml) was used as a positive control for each tested population. In all instances, 2.5 x 10^5 cells could only be plated in a single well per condition, given the limited number of available cells from frozen stocks. IFNγ-producing cells were enumerated by ELISPOT and data are represented as the unstimulated-corrected IFNγ spot-forming cells per 2.5 x 10^5 cells. Each pig is represented as a distinct symbol. For each population of all the tested pigs, more than 200 IFNγ spots were obtained with ConA stimulation (too many to count in most instances) indicating that our experimental conditions (cryopreservation and magnetic sorting) did not affect the ability of cells to secrete IFNγ.

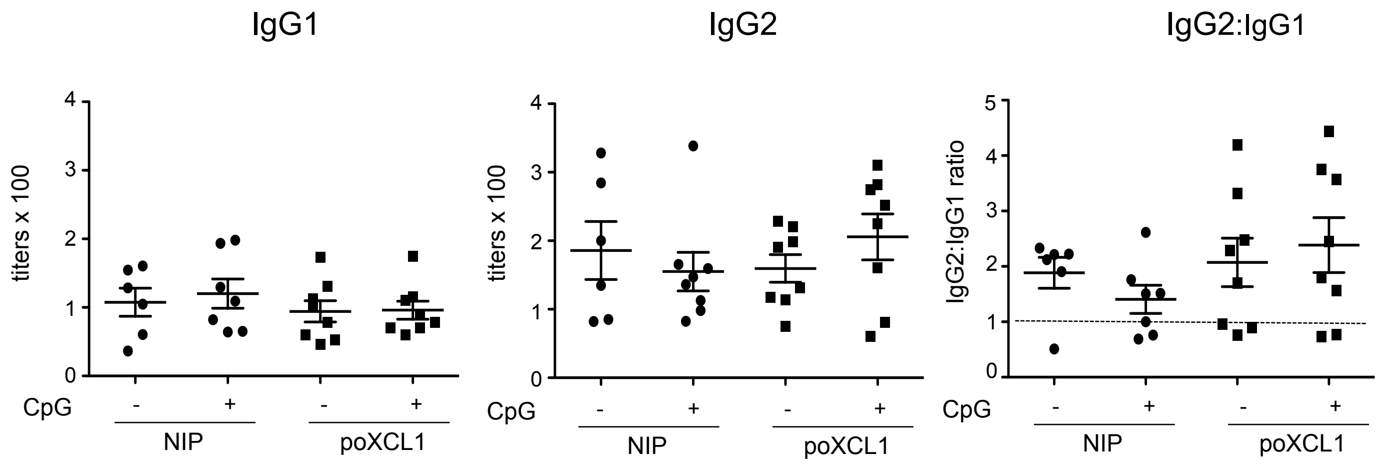

Supplementary Fig. 3. Pre-existing anti-M2e IgG1 and IgG2 titers at the time of immunization in experiment 2. The IgG1 and IgG2 anti-M2e titers in piglets' sera were measured by ELISA at D0 and are shown for the different groups. Titers were similar across groups. The IgG2 titer : IgG1 titer ratios are depicted. Arithmetic means and standard errors of the mean are shown.
